# Implementing a Physical Health Clinic on an Acute Adult Inpatient Psychiatric Ward

**DOI:** 10.1192/bjo.2022.287

**Published:** 2022-06-20

**Authors:** Rowan De Souza, Alisha Patel

**Affiliations:** St George's and South West London & St George's Mental Health NHS Trust, London, United Kingdom

## Abstract

**Aims:**

Acute Inpatient Psychiatric Wards present the challenge of a high turnover of patients who have multiple physical health comorbidities that both contribute to patients’ overall morbidity and may exacerbate any mental illness. Furthermore, there are a number of physical health parameters which must be checked and monitored in the initiation of psychiatric treatments. It is therefore important that patients receive physical examination, blood tests and Electrocardiograms (ECGs). The busy environment of inpatient units and the acute presentation of patients, who often decline interventions, lack capacity or cannot communicate their physical health problems, mean these assessments are often missed when offered in an ad hoc fashion. This Quality Improvement Project looked at implementing a Physical Health Clinic to look at whether this structured environment would provide better coverage of these physical health assessments.

**Methods:**

The number of physical examinations, blood tests and ECGs both offered but declined and successfully obtained was measured on an Inpatient Ward with 20 patients and 2 junior doctors over 2 weeks with assessments being offered in an ad hoc fashion. Following this, a structured clinic run by a doctor and nurse with three 20 minute appointments three times a week was implemented and the same data collected over 2 weeks. A paired T-test was used to evaluate the results.

**Results:**

There was a statistically significant increase in the number of successfully obtained physical examinations, bloods tests and ECGs when the Physical Health Clinic was implemented (Mean difference = 7.33, Two tailed P value = 0.0480,95% confidence interval 0.16–14.50, t = 4.4, df = 2, standard error of difference = 1.667). However, there was no difference between the number of bloods, examinations and ECGs offered but declined (Mean difference = 4.83, Two tailed P value = 0.2495, 95% confidence interval −3.92–8.58, t = 1.6059, df = 2, standard error of difference = 1.453).

**Conclusion:**

The clinic led to a statistically significant increase in the number of examinations, blood tests and ECGs successfully obtained. The reasons for this are hypothesized that having a structured clinic prepares the patient to have a physical assessment and ensures their availability, provides motivation for staff and increases the efficiency of assessments with appropriate teamwork between doctors and nurses. Issues with the Clinic are limited availability of junior doctor and nursing staff and emergencies disrupting the functioning of the clinic. Implementing the clinic on a wider scale is required to assess its effectiveness.

